# Supplementary material for: Managing hypertension in frail oldest-old—The role of guideline use by general practitioners from 29 countries
Source: PLoS One. 2020 Jul 10;15(7):e0236064. doi: 10.1371/journal.pone.0236064 (PMC7351187; doi:10.1371/journal.pone.0236064)
Supplement: S1 Appendix — (PDF) [file pone.0236064.s001.pdf]

S1 Appendix. Names of the most frequent guidelines mentioned (n=798)

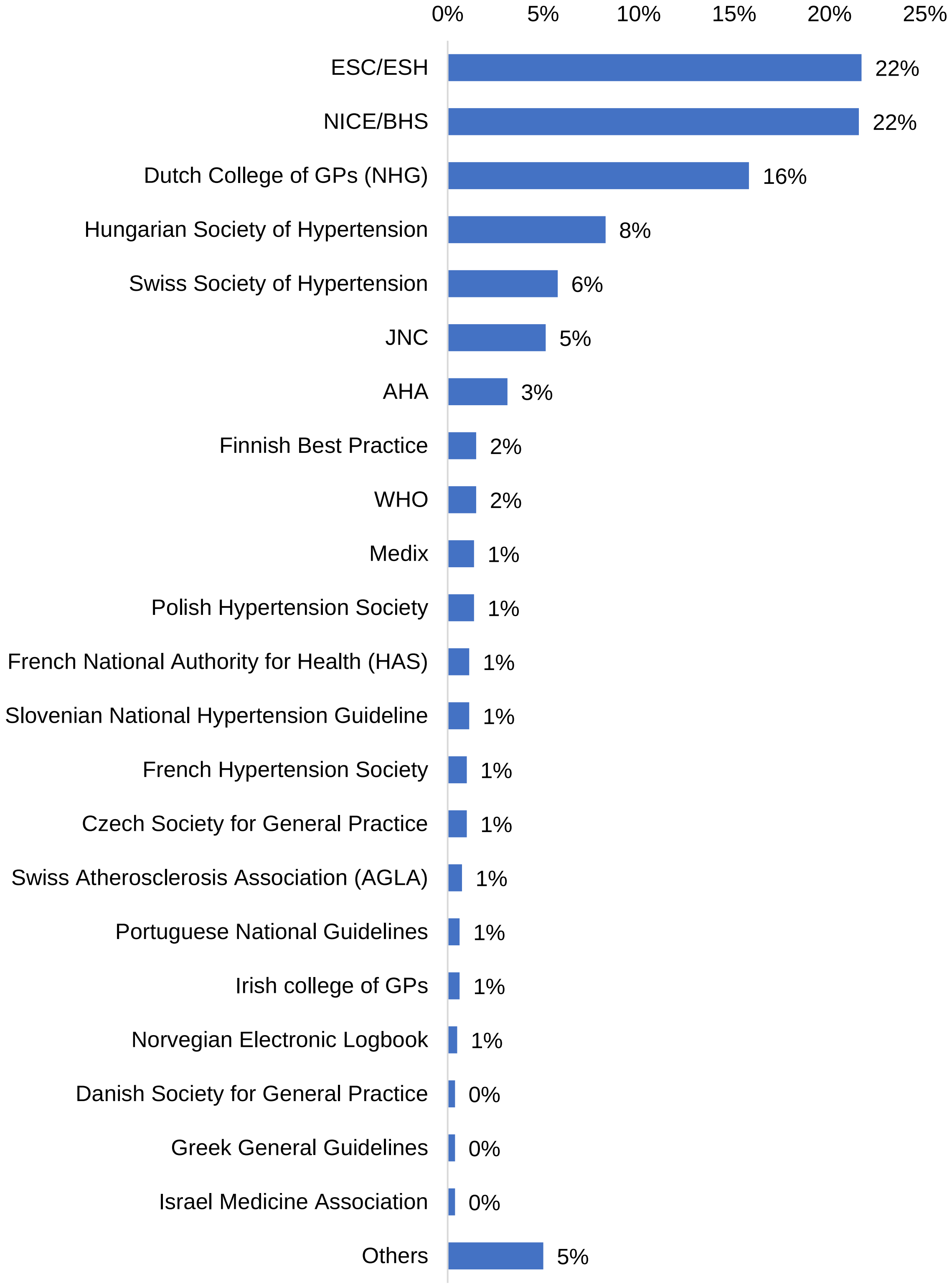

AHA: American Heart Association; BHS: British Hypertension Society; ESC: European society of cardiology; ESH: European Society of Hypertension; JNC: Joint National Committee; Medix: Large Swiss GP network's own guideline; NICE: National Institute for Health and Care Excellence; WHO: World Health Organization.
