# Supplementary material for: Managing hypertension in frail oldest-old—The role of guideline use by general practitioners from 29 countries
Source: PLoS One. 2020 Jul 10;15(7):e0236064. doi: 10.1371/journal.pone.0236064 (PMC7351187; doi:10.1371/journal.pone.0236064)
Supplement: S2 Appendix — (PDF) [file pone.0236064.s002.pdf]

S2 Appendix. Percentage of guideline use per country

Country	Percentage of guidelines use (%)
Brazil	85,7
Ukraine	88.4
Slovenia	87.5
Greece	87.0
Czech Republic	81.5
Macedonia	81.0
Romania	80.0
Sweden	76.5
Finland	75.0
Portugal	72.6
Bosnia Herzegovina	57,7
Spain	61.4
Israel	60.7
Poland	59.4
Hungary	58.7
Ireland	57.6
Switzerland	56.1
Netherlands	55.7
Norway	51.6
Latvia	51.1
Austria	46,4
Denmark	50.0
United Kingdom	50.0
France	47.6
Italy	47.4
Germany	44.8
Luxembourg	42.9
Turkey	29.4
New Zealand	25.6
