# Supplementary material for: Managing hypertension in frail oldest-old—The role of guideline use by general practitioners from 29 countries
Source: PLoS One. 2020 Jul 10;15(7):e0236064. doi: 10.1371/journal.pone.0236064 (PMC7351187; doi:10.1371/journal.pone.0236064)
Supplement: S3 Appendix — (DOCX) [file pone.0236064.s003.docx]

**S3 Appendix. Hypertension treatment recommendations for frail oldest-old and oldest-old in the three most mentioned guidelines**

|  | **Frail oldest-old patients** | | | | |  | **Oldest-old patients** | | | | |
| --- | --- | --- | --- | --- | --- | --- | --- | --- | --- | --- | --- |
|  | **SBP 140 mmHg** | |  | **SBP 160 mmHg** | |  | **SBP 140 mmHg** | |  | **SBP 160 mmHg** | |
| **Treatment is recommended**^1^ |  |  |  |  |  |  |  |  |  |  |  |
| **ESC 2013** | No |  |  | - ^2^ |  |  | No |  |  | Yes |  |
| **NICE 2013** | No |  |  | - ^2^ |  |  | No |  |  | Yes |  |
| **NHG 2012** | No |  |  | - ^2^ |  |  | No |  |  | No |  |

^1^ Current recommendations at the time of the study (2016)

^2^ Decision left to the treating physician

References

1. Mancia G, Fagard R, Narkiewicz K, et al. 2013 ESH/ESC Guidelines for the management of arterial hypertension: the Task Force for the management of arterial hypertension of the European Society of Hypertension (ESH) and of the European Society of Cardiology (ESC). J Hypertens. 2013;31(7):1281‐1357.
2. Jaques H; National Institute for Health and Clinical Excellence (NICE). NICE guideline on hypertension. Eur Heart J. 2013;34(6):406‐408.
3. Wiersma T, Smulders YM, Stehouwer CD, Konings KT, Lanphen J. [Summary of the multidisciplinary guideline on cardiovascular risk management (revision 2011)].
